# Editorial: The role of glia in ataxia and other neurodegenerative disorders

**DOI:** 10.3389/fncel.2026.1909135

**Published:** 2026-06-30

**Authors:** Milica Cerovic, Rosella Abeti

**Affiliations:** 1 Department of Neuroscience, Mario Negri Institute for Pharmacological Research IRCCS, Milan, Italy; 2 Ataxia Centre, Department of Clinical and Movement Neurosciences, University College London (UCL) Queen Square Institute of Neurology, London, United Kingdom; 3 School of Life and Health Sciences, University of Roehampton, London, United Kingdom

**Keywords:** ataxia, glia, neurodegeneration, neuroinflammation, neurons

Neurodegenerative disorders have traditionally been viewed through a neuron-centric lens, with disease onset and progression attributed primarily to neuronal dysfunction and loss. Over the past decades, however, advances in cellular and molecular neuroscience have transformed our current understanding of these conditions, shifting the focus toward a broader consideration of the involvement of multiple cell types. It is now evident that glial cells—including astrocytes, microglia, oligodendrocytes, and other brain-resident immune populations—play critical roles not only in maintaining central nervous system homeostasis but also in driving, modulating, exacerbating, and potentially limiting neurodegenerative processes.

This Research Topic, *The Role of Glia in Ataxia and Other Neurodegenerative Disorders*, brings together studies that highlight the multifaceted contributions of glial cells across a spectrum of neurological diseases. Collectively, these articles reinforce the concept that neurodegeneration arises from complex interactions between neurons and their surrounding cellular environment, and that glial cells represent promising therapeutic targets for future interventions.

One emerging theme is that glial dysfunction may occur at the earliest stages of disease and contribute directly to regional vulnerability. In their study of spinocerebellar ataxia type 3 (SCA3), Emerson et al. provide compelling transcriptomic evidence that the spinal cord is an early and previously underappreciated site of pathology. Their findings reveal alterations in inflammatory pathways, lipid metabolism, and oligodendrocyte-associated genes, suggesting that disturbances in glial homeostasis precede or accompany neuronal degeneration. Particularly notable is the identification of oligodendrocyte-related transcriptional and splicing abnormalities, supporting the growing view that white matter and myelin-associated pathology are important contributors to disease progression in ataxias.

The complexity of glial responses is further illustrated by Jeong et al., who investigated the role of astrocytic Toll-like receptor 3 (TLR3) signaling in rodent models of Parkinson's disease. Although neuroinflammation is often considered detrimental, their work demonstrates that activation of TLR3 in astrocytes can exert neuroprotective effects by promoting the production of neurotrophic factors and attenuating degeneration of the nigrostriatal pathway. These findings show that glial activation is shaped by the surrounding pathological environment, challenging the simplistic classification of glial responses as either beneficial or harmful. Instead, glial phenotypes exist along a dynamic spectrum influenced by disease stage, local environment, and signaling pathways.

The importance of neuron-glia communication is also emphasized by Orsini et al., who examined the effects of Small ubiquitin-related modifier 2 (SUMO2) on Tau-mediated pathology. Their study demonstrates that expression of the pathogenic TauP301L mutation (one of the best-characterized causes of inherited frontotemporal dementia with Parkinsonism linked to chromosome 17) not only damages neurons but also induces toxicity in astrocytes and microglia. Remarkably, enhancing SUMO2 expression protected both neuronal and glial populations, revealing a shared vulnerability and a potentially conserved protective mechanism. These findings underscore the notion that glial pathology is not merely a secondary consequence of neuronal dysfunction but may actively shape disease outcomes.

Beyond individual glial populations, understanding the diversity and plasticity of the brain's immune landscape remains a major challenge. The comprehensive review by Zhao et al. expands this perspective by examining brain-resident macrophages, including microglia and border-associated macrophages. The authors describe how these cells contribute to both tissue protection and disease progression across a range of neurodegenerative and neurological disorders. Importantly, they discuss emerging strategies aimed at reprogramming glial immune responses to enhance repair while limiting chronic neuroinflammation. Such approaches may offer new opportunities to transform endogenous immune cells from drivers of pathology into therapeutic allies.

Collectively, the contributions to this Research Topic highlight the expanding role of glial cells in neurodegenerative disorders. The studies presented here reveal how alterations in oligodendrocytes, astrocytes, microglia, and brain-resident macrophages contribute to disease processes ranging from ataxia, Parkinson's disease to tauopathies. Importantly, several of these changes emerge at early stages of pathology, highlighting glial dysfunction as a potential source of both biomarkers and therapeutic targets.

Beyond their disease-specific findings, these articles emphasize a common challenge for the field: understanding the molecular mechanisms that shape glial responses, including how these cells support tissue homeostasis, and how loss of homeostatic function contributes to neurodegeneration. Addressing this question will require integrating emerging approaches, including single-cell and spatial technologies, with functional studies that capture the complexity of neuron-glia interactions *in vivo*.

As our understanding of glial biology continues to evolve, it is becoming increasingly clear that effective therapies for neurodegenerative disorders may depend not only on protecting vulnerable neurons, but also on harnessing the diverse and context-dependent functions of glial cells. The studies collected in this Research Topic provide important insights toward this goal and highlight new directions for future research.

